# Diagnostic utility of the modified inflammatory burden index and PCT/LYM ratio for sepsis stratification and early prediction of septic shock

**DOI:** 10.3389/fcimb.2026.1845721

**Published:** 2026-10-07

**Authors:** Zhen-Zhen Ding, Qian Li, Chun-Yan Ma, Hang Li

**Affiliations:** 1 Department of Laboratory Medicine, Kaifeng Central Hospital, Kaifeng, Henan, China; 2 Key Laboratory of Tumor Biological Marker Detection of Kaifeng, Kaifeng, Henan, China

**Keywords:** biomarker, diagnostic efficacy, modified inflammatory burden index, sepsis, septic shock

## Abstract

**Objective:**

This study aimed to investigate the diagnostic performance of a modified inflammatory burden index (mIBI) and conventional biomarkers in sepsis and septic shock and assess the clinical utility of their combined application with the PCT/LYM ratio.

**Methods:**

A retrospective analysis included 45 patients with sepsis, 77 with septic shock, and 54 with non-septic infectious inflammation (control). Levels of procalcitonin (PCT), C-reactive protein (CRP), interleukin-6 (IL-6), neutrophil count (NEU), and lymphocyte count (LYM) were measured. Composite indices including mIBI, PCT/LYM, CRP/LYM, and NLR were calculated. Multivariable logistic regression evaluated the independent diagnostic value of mIBI and PCT/LYM after adjusting for confounders.

**Results:**

Inflammatory markers and composite indices increased progressively from control to sepsis to septic shock, whereas LYM decreased (all *P* < 0.05). For sepsis diagnosis, PCT/LYM showed strong diagnostic performance (AUC = 0.851, 95% CI: 0.775–0.928; sensitivity 80.00%, specificity 81.48% at cutoff 0.91), followed by mIBI (AUC = 0.806, specificity 88.89%). For septic shock, PCT/LYM (AUC = 0.880) and mIBI (AUC = 0.877) were comparable to PCT and IL-6 (all AUC = 0.879). Multivariable analysis revealed that after adjustment for age, sex, qSOFA, diabetes, and renal insufficiency, both log_10_(mIBI) (OR = 2.771, P < 0.001) and PCT/LYM (OR = 1.045, P = 0.002) were independent predictors for sepsis. For septic shock, log_10_(mIBI) retained independent predictive capacity (OR = 1.399, P = 0.030), whereas PCT/LYM showed a borderline association (OR = 1.004, P = 0.053).

**Conclusion:**

The composite framework integrating PCT/LYM and mIBI addresses limitations of single biomarkers. PCT/LYM demonstrated independent predictive value, with a numerically higher AUC than PCT alone for both sepsis and septic shock, although the direct DeLong comparison did not reach statistical significance, supporting its potential utility as part of a composite screening approach. Modified IBI showed strong independent diagnostic value for sepsis and high univariate discriminative ability for septic shock. These routinely available indicators offer timely, cost-effective advantages for early sepsis identification and risk stratification.

## 1 Introduction

Sepsis is a life-threatening condition characterized by organ dysfunction resulting from a dysregulated host immune response to infection. It represents a major global public health concern in critical care medicine. According to data from the Global Burden of Disease study, the incidence of sepsis continues to increase, with mortality rates in intensive care units (ICUs) reaching approximately 25% to 30% (Rudd et al., 2020). Based on the Sepsis-3 international consensus, mortality among patients with septic shock may reach 40% to 60%, with an estimated increase of 7.6% in mortality risk for each hour of delay in the initiation of effective antimicrobial therapy (Singer et al., 2016; Evans et al., 2021). Early and accurate identification, followed by timely intervention, remains central to improving clinical outcomes in individuals with sepsis. However, the clinical presentation is often heterogeneous and nonspecific, and existing clinical scoring systems and single biomarkers are insufficient to meet the demands of precise diagnosis.

Procalcitonin (PCT) is an FDA-cleared biomarker indicated as an adjunct in the assessment of sepsis and lower respiratory tract infections, as well as in guiding antibiotic de-escalation, rather than as a stand-alone diagnostic criterion. It has been widely used to support clinical judgment in sepsis management and in the identification of bacterial infections. However, PCT levels may also be elevated in non-infectious inflammatory conditions, including postoperative states, trauma, and malignancy, thereby limiting its specificity (Ito et al., 2022). Similarly, conventional biomarkers such as C-reactive protein (CRP) and interleukin-6 (IL-6), although readily measurable, lack sufficient specificity and have limited value in early diagnosis (Carcò et al., 2022; Cajander et al., 2024). Lymphocyte count (LYM), which reflects immune status, is influenced by multiple factors and does not reliably represent immune function in sepsis (Samuelsen et al., 2024).

From a pathophysiological perspective, sepsis is characterized by a complex imbalance involving infection, inflammation, and immune response. Single biomarkers are therefore insufficient to comprehensively reflect this multifaceted process. In recent years, composite biomarkers have demonstrated significant potential for improving diagnostic precision by integrating multidimensional pathological signals and quantifying multiple underlying mechanisms. For example, the PCT/LYM ratio enables the combined assessment of bacterial burden and immune suppression, while a modified inflammatory burden index (mIBI) incorporates infection specificity, inflammatory intensity, and the degree of immunosuppression into a single metric, where NEU was replaced by PCT to incorporate infection-specific information.

To the best of our knowledge, extensive data search reveals no prior systematic evaluation of these specific composite indices for sepsis stratification. This study systematically evaluates the diagnostic performance of composite indices, including mIBI and PCT/LYM, in comparison with traditional biomarkers for the early identification of sepsis and early prediction of septic shock. The mIBI was originally defined as CRP × NEU/LYM (i.e., CRP × NLR) in the published literature (Xie et al., 2022); the present study adapted this index by substituting PCT for NEU to enhance infection specificity, yielding the formula: mIBI = PCT × CRP/LYM, in which neutrophil count was replaced by PCT to incorporate infection-specific information. This modification is consistent with our team’s previous work (Li et al., 2026). In addition, it aims to clarify their clinical utility in disease severity stratification, thereby providing a reliable laboratory basis for precision-oriented clinical management.

The modified IBI formula (mIBI = PCT × CRP/LYM) was conceptualized in our team’s preliminary exploratory study (Li et al., 2026), which reported univariate ROC findings on PCT/LYM and IBI in a single-center Chinese cohort (n = 132, enrollment October 2020-May 2025). The present study represents a substantial methodological and temporal extension of that clinical population: the cohort was expanded to n = 176 (enrollment through December 2025), and novel analyses including multivariable logistic regression with sensitivity analyses, DeLong pairwise AUC comparisons, 1000-iteration bootstrap internal validation, and head-to-head benchmarking against qSOFA were performed. These methodologically advanced analyses were not reported in the prior publication, which was limited to univariate diagnostic cutoffs. The two manuscripts address distinct analytical questions and are directed at different scientific audiences.

## 2 Study participants and methods

### 2.1 Study participants

This retrospective study included 122 patients clinically diagnosed with sepsis at Kaifeng Central Hospital between October 2020 and December 2025. Blood routine parameters and inflammatory marker data obtained within 24 hours of admission were collected. Inclusion criteria were based on the *International Guidelines for Diagnosis and Treatment of Sepsis and Septic Shock (2021 Edition)*. Patients were further classified into a sepsis group (*n* = 45) and a septic shock group (*n* = 77).

Exclusion criteria included the presence of autoimmune diseases, malignant tumors, or severe hepatic and renal insufficiency; patients within 48 hours after surgery or in the acute phase of trauma; and those with incomplete clinical data.

In addition, 54 patients with non-septic infectious inflammation admitted during the same period were selected as the control group and were diagnosed according to internationally recognized diagnostic criteria for systemic inflammatory response syndrome (SIRS) (Joseph and Nasir, 2025). To ensure the quality of the control group, strict exclusion criteria were applied: patients with confirmed or suspected bacterial or viral infections, elevated PCT levels (>0.5 ng/mL) suggestive of occult infection, malignancy, autoimmune diseases, recent major surgery (within 2 weeks), or immunosuppressive therapy were excluded. All control patients were clinically confirmed to have non-infectious systemic inflammatory conditions by attending physicians based on comprehensive clinical assessment, laboratory findings, and imaging results. This study was approved by the Ethics Committee of Kaifeng Central Hospital (2025ks-1w048).

### 2.2 Methods

#### 2.2.1 Data collection

Electronic medical records were reviewed, and the following data were collected: baseline characteristics at admission, including age, sex, body mass index (BMI), systolic blood pressure (SBP), and diastolic blood pressure (DBP); medical history, including diabetes mellitus, hypertension, hepatitis, renal insufficiency, chronic tracheitis, and pulmonary emphysema; and laboratory parameters at admission, including PCT, CRP, IL-6, NEU, and LYM.

#### 2.2.2 Laboratory testing

Serum PCT and IL-6 levels were measured using the Roche E801 electrochemiluminescence (ECL) system and corresponding reagents (reference ranges: PCT < 0.05 ng/mL; IL-6 < 7.00 pg/mL). CRP levels were determined by immunoturbidimetry (reference range: < 10 mg/L). Neutrophil count (NEU) and LYM were measured using the Sysmex XN9000 hematology analyzer and supporting reagents [reference ranges: NEU (2.0-7.0) × 10^9^/L; LYM (0.8-4.0) × 10^9^/L]. The modified IBI (mIBI) was calculated as follows: mIBI = PCT × CRP/LYM, in which neutrophil count was replaced by PCT to incorporate infection-specific information. The original IBI was defined as CRP × NEU/LYM (i.e., CRP × NLR), integrating inflammatory intensity (CRP), neutrophil activation (NEU), and lymphocyte depletion (LYM) into a single metric. In the present study, PCT was substituted for NEU to enhance infection specificity, given that PCT is more specific to bacterial infection than neutrophil count. Mathematically, since PCT and CRP are in the numerator and LYM is in the denominator, the formula captures the triad of infection burden (PCT↑), inflammatory amplification (CRP↑), and immunosuppression (LYM↓). For each participant, mIBI was calculated from that individual’s PCT, CRP and LYM values before any group-level analysis; group medians of the three components were not combined to derive mIBI, because the median of a non-linear ratio does not equal the ratio of component medians. As expected from this individual-level construction, mIBI increased progressively from control through sepsis to septic shock.

### 2.3 Statistical analysis

Statistical analyses were performed using SPSS version 26.0, and graphical outputs were generated using GraphPad Prism version 9.5. Categorical variables were expressed as counts and percentages [*n* (%)] and compared using the chi-squared (*χ²*) test. Continuous variables were first assessed for normality using the Shapiro–Wilk test. Normally distributed data were expressed as mean ± standard deviation (*x̄* ± *s*) and compared using one-way analysis of variance (ANOVA), followed by pairwise comparisons using the LSD t-test. Non-normally distributed data were expressed as median (interquartile range) [M(Q1, Q3)] and compared using the Kruskal–Wallis H test, with pairwise comparisons conducted using Dunn’s test with Bonferroni correction.

Receiver operating characteristic (ROC) curves were constructed, and the area under the curve (AUC) and corresponding 95% confidence intervals (95% CI) were calculated. Optimal cutoff values were determined using the Youden index to evaluate diagnostic sensitivity and specificity, as well as the diagnostic performance of each indicator for sepsis and septic shock. AUCs were compared using the DeLong test. The family of comparisons for Bonferroni correction was prospectively defined as eight pairwise comparisons per ROC diagnostic panel (Panel A: control vs. sepsis, n=99; Panel B: control vs. septic shock, n=131), in which mIBI was compared with each of the eight alternative biomarkers (PCT, CRP, IL-6, LYM, NEU, CRP/LYM, PCT/LYM, and NLR). Importantly, no broader all-pairs comparisons among all nine biomarkers were performed; the statistical testing plan was restricted to these eight pre-specified comparisons per panel. This yielded a corrected significance threshold of α = 0.05/8 = 0.00625 for each panel. *Post hoc* comparisons involving qSOFA and the direct PCT/LYM vs. PCT comparison were considered exploratory and were not included in the primary comparison family. Multivariable logistic regression analysis was performed to evaluate the independent diagnostic value of log_10_-transformed mIBI and PCT/LYM after adjusting for age, sex, qSOFA score, history of diabetes mellitus, and history of renal insufficiency. LYM was not included as a covariate in the primary models because it is a denominator component of both mIBI (mIBI = PCT × CRP/LYM) and PCT/LYM (PCT/LYM = PCT/LYM); simultaneous inclusion of a composite ratio and one of its constituent variables introduces mathematical coupling that can bias coefficient estimation and complicate interpretation, even when variance inflation factors are low. To assess whether this omission influenced conclusions, a supplementary model additionally adjusting for LYM was fitted as a sensitivity analysis. qSOFA was retained as a covariate because it is assessed at presentation before diagnosis and serves as a readily available bedside clinical marker of illness severity that may confound the biomarker–diagnosis association; to evaluate whether adjustment for qSOFA over-corrected the association of interest, a parallel sensitivity model excluding qSOFA was also fitted. Due to the markedly right-skewed distribution of mIBI (skewness > 3), log_10_ transformation was applied to approximate normality prior to parametric testing and regression analysis. Two models were constructed for each diagnostic comparison: Model 1 incorporated log_10_(mIBI), and Model 2 incorporated PCT/LYM. Model performance was evaluated by the area under the ROC curve, Nagelkerke R², and Hosmer–Lemeshow goodness-of-fit test. Multicollinearity was assessed using the variance inflation factor (VIF). Internal validation of model AUCs was performed using 1000 bootstrap resamples. A two-sided p value < 0.05 was considered statistically significant.

This study addressed four distinct diagnostic questions: (i) discrimination of combined sepsis/septic shock from non-septic inflammation (Control vs. Sepsis+Shock, n=176) by multivariable logistic regression; (ii) discrimination of sepsis alone from non-septic inflammation (Control vs. Sepsis, n=99) by ROC analysis; (iii) discrimination of septic shock alone from non-septic inflammation (Control vs. Shock, n=131) by ROC analysis; and (iv) prediction of septic shock progression among sepsis patients (Sepsis vs. Shock, n=122) by multivariable logistic regression. These analyses involve different patient subsets and clinical endpoints and are reported separately.

## 3 Results

### 3.1 Clinical data of the three groups

In this study, baseline clinical characteristics were comparable among the control group (*n* = 54), sepsis group (*n* = 45), and septic shock group (*n* = 77) (see **Table 1**). No statistically significant differences were observed among the three groups in terms of sex distribution, age, BMI, SBP, DBP, history of hypertension, history of hepatitis, or history of chronic tracheitis and pulmonary emphysema (all *p* > 0.05). However, the qSOFA score showed a statistically significant difference among the three groups (*χ²* = 30.698, *p* < 0.001), with progressively higher values from the control group (0.80 ± 0.83) to the sepsis group (0.96 ± 0.95) and the septic shock group (1.57 ± 0.97). Regarding blood culture results, all sepsis and septic shock patients had positive blood cultures, while all control patients were confirmed as blood culture negative after quality control review. Regarding underlying comorbidities, a statistically significant difference was identified in the prevalence of renal insufficiency among the three groups (*χ²* = 10.075, *P =* 0.006). The proportion of patients with a history of renal insufficiency was 9.3% in the control group, 20.00% in the sepsis group, and 32.47% in the septic shock group, indicating a potential association between renal insufficiency and the progression of sepsis. Although no statistically significant difference was observed in the prevalence of diabetes mellitus (*χ²* = 5.299, *P =* 0.071), a higher proportion was noted in the sepsis group (44.44%) and septic shock group (40.26%) compared with the control group (24.1%), indicating a possible association between diabetes mellitus and increased susceptibility to sepsis. All sepsis and septic shock patients had positive blood cultures, while all control patients had negative blood culture results. Among culture-positive patients, Gram-negative bacteria were the predominant pathogens in both the sepsis group (36/45, 80.0%) and the septic shock group (59/77, 76.6%), followed by Gram-positive bacteria (8/45, 17.8% vs. 15/77, 19.5%), mixed infection with both Gram-positive and Gram-negative bacteria (1/45, 2.2% vs. 2/77, 2.6%), and fungus (0/45, 0.0% vs. 1/77, 1.3%).

**Table 1:** Comparison of baseline clinical characteristics among the three groups.

General data	Control group (*n* = 54)	Sepsis group (*n* = 45)	Septic shock group (*n* = 77)	*χ^2^* value	*p* value
Sex (male/female)	26/28	25/20	53/24	5.930	0.052
Age (year)	72.44 ± 14.10	69.53 ± 17.97	72.44 ± 13.86	10.587	0.330
BMI (kg/m^2^)	23.59 ± 1.61	23.82 ± 3.85	23.97 ± 5.28	3.132	0.117
SBP (mmHg)	118.36 ± 21.53	125.40 ± 18.62	128.53 ± 22.38	1.327	0.507
DBP (mmHg)	82.19 ± 8.74	86.12 ± 8.98	88.31 ± 9.79	1.382	0.674
qSOFA	0.80 ± 0.83	0.96 ± 0.95	1.57 ± 0.97	30.698	<0.001
History of diabetes mellitus (n, %)	13 (24.1)	20 (44.44)	31 (40.26)	5.299	0.071
History of hypertension (n, %)	22 (40.7)	29 (64.44)	52 (67.53)	1.492	0.474
History of hepatitis (n, %)	3 (5.6)	8 (17.78)	16 (20.78)	5.940	0.051
History of renal insufficiency (n, %)	5 (9.3)	9 (20.00)	25 (32.47)	10.075	0.006
History of chronic tracheitis and pulmonary emphysema (n, %)	10 (18.5)	11 (24.44)	19 (24.68)	0.787	0.675
Gram-negative bacteria (n, %)	/	36 (80.0%)	59 (76.6%)	0.686	0.877
Gram-positive bacteria (n, %)	/	8 (17.8%)	15 (19.5%)		
Mixed infection (n, %)	/	1 (2.2%)	2 (2.6%)		
Fungus (n, %)	/	0 (0.0%)	1 (1.3%)		

### 3.2 Inflammatory and immune indicator levels in the three groups

Statistically significant differences were observed in the levels of PCT, CRP, IL-6, LYM, CRP/LYM, PCT/LYM, mIBI, and neutrophil-to-lymphocyte ratio (NLR) among the control, sepsis, and septic shock groups (all *p* < 0.05), whereas no statistically significant difference in NEU levels was identified among the three groups (*P =* 0.224). The levels of inflammatory markers and composite indices demonstrated a progressive increase in the order of control < sepsis < septic shock, while LYM levels exhibited an inverse decreasing trend across these groups. Among the evaluated indicators, mIBI demonstrated the most pronounced differences among groups (H = 58.820, *p* < 0.001). Specifically, the median mIBI value in the septic shock group [3157.04 (130.34, 33612.60)] was approximately 183-fold higher than that in the control group [17.22 (3.47, 49.26)]. Detailed results are presented in **Table 2**, **Figure 1** presents the distribution of indicator levels across the three groups using box plots.

**Table 2:** Comparison of inflammatory and immune biomarkers among the three groups.

Indicator	Control group (*n* = 54)	Sepsis group (*n* = 45)	Septic shock group (*n* = 77)	*H* value	*p* value
PCT (ng/mL)	0.23 (0.11,0.58)^b^	4.14 (1.01,24.46)^a^	16.72 (1.79,54.33)^a^	61.350	0.000
CRP (mg/L)	52.12 (24.22,138.06)	115.23 (25.95,198.88)	177.46 (98.50,294.26)^a^	28.376	0.000
IL-6 (pg/mL)	58.71 (21.70,132.48)^b^	557.70 (170.30,3940.00)^a^	920.20 (179.75,5000.00)^ab^	62.672	0.000
NEU (×10^9^/L)	8.26 (5.48,12.73)	10.66 (6.85,14.01)	11.40 (4.56,16.08)	2.991	0.224
LYM (×10^9^/L)	1.02 (0.63,1.40)^b^	0.56 (0.41,0.75)^a^	0.60 (0.31,1.08)^a^	17.706	0.000
CRP/LYM	47.13 (23.78,135.42)^b^	199.71 (38.48,379.12)^a^	345.59 (81.78,567.95)^a^	28.929	0.000
PCT/LYM	0.25 (0.09,0.86)^b^	5.33 (1.38,50.13)^a^	23.89 (1.79,128.21)^a^	62.293	0.000
mIBI	17.22 (3.47,49.26)^b^	703.75 (49.14,5599.58)^a^	3157.04 (130.34,33612.60)^a^	58.820	0.000
NLR	8.02 (5.07,15.20)^b^	18.22 (10.72,30.27)^a^	18.83 (8.16,29.36)^a^	18.825	0.000

^a^indicates that *vs* the control group, *p* < 0.05; ^b^indicates that *vs* the sepsis group, *p* < 0.05.

**Figure 1:**
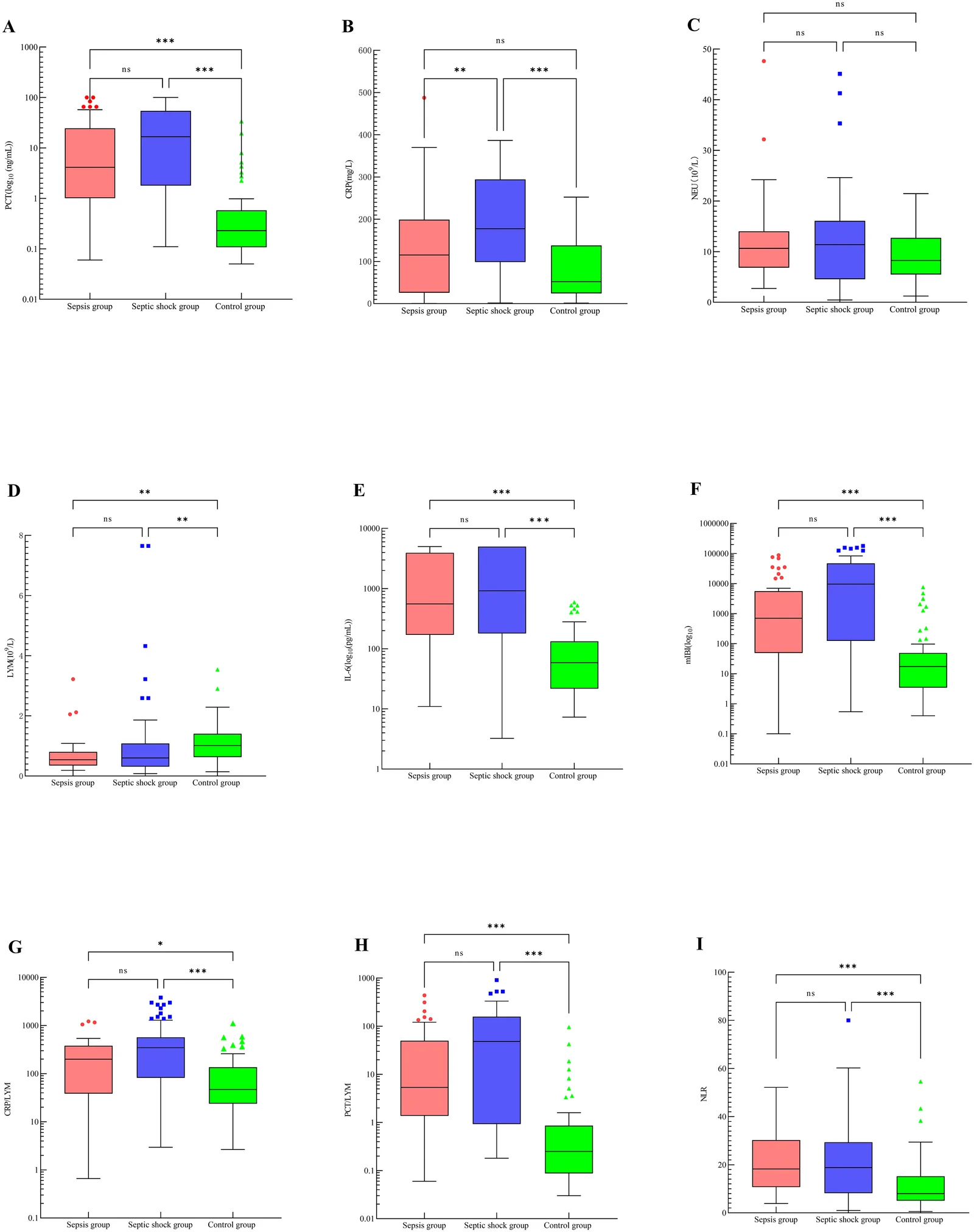
Distribution of inflammatory and immune biomarkers among the control, sepsis, and septic shock groups. Red box body and scattered points = sepsis group (n = 45), blue box body and scattered points = septic shock group (n = 77), green box body and scattered points = control group (n = 54); box body: the upper and lower edges represent the 75th and 25th percentiles of the data respectively (interquartile range, IQR); the center line of the box body represents the median of the data; error bar: extending to the minimum and maximum values of this set of data; scattered points: corresponding to the original detection value of a single sample in each group. Comparison among groups was conducted by Dunn multiple comparison after the Kruskal-Wallis test. “ns” indicates that there were no statistically significant differences among the groups (p ≥ 0.05); “*” indicates that there were statistically significant differences among the groups (p < 0.05); “***” indicates that there were highly statistically significant differences among the groups (p < 0.001). Panels **(A–I)** correspond to the following serum biological indicators in sequence: **(A)** indicates procalcitonin (PCT), **(B)** indicates C-reactive protein (CRP), **(C)** indicates neutrophil count (NEU), **(D)** indicates lymphocyte count (LYM), **(E)** indicates interleukin-6 (IL-6), **(F)** indicates mIBI, **(G)** indicates CRP/LYM, **(H)** indicates PCT/LYM, and **(I)** indicates NLR.

### 3.3 Multivariable logistic regression analysis

In the sepsis diagnostic model (**Table 3**, panel A, Control Group vs Sepsis + Septic Shock Group, n = 176), log_10_(mIBI) was identified as an independent predictor of sepsis (OR = 2.771, 95% CI: 1.920–3.999, P < 0.001). In the alternative model, PCT/LYM similarly emerged as an independent predictor (OR = 1.045, 95% CI: 1.016–1.076, P = 0.002). The model incorporating log_10_(mIBI) yielded an AUC of 0.893, which was higher than that of the model incorporating PCT/LYM (AUC = 0.859), indicating that mIBI may possess stronger discriminative ability for sepsis diagnosis. Sensitivity analyses excluding qSOFA yielded consistent results (mIBI: OR = 2.858, 95% CI: 1.979–4.127, P < 0.001, AUC = 0.881; PCT/LYM: OR = 1.047, 95% CI: 1.016–1.079, P = 0.003, AUC = 0.846), confirming that the independent predictive value of both composite indices was not driven by qSOFA adjustment. Supplementary models additionally adjusting for LYM also produced consistent findings (mIBI: OR = 2.784, P < 0.001; PCT/LYM: OR = 1.044, P = 0.004), with LYM itself non-significant in both models (P = 0.927 and P = 0.583, respectively).

**Table 3:** Multivariate logistic regression analysis for the diagnosis of sepsis (control group vs sepsis + septic shgck Group, n = 176).

Variable	Model 1 OR (95% CI)	Model 1 *P*	Model 2 OR (95% CI)	Model 2 *P*
Age	0.993 (0.965-1.022)	0.636	0.992 (0.965-1.019)	0.555
Female (vs Male)	0.746 (0.315-1.765)	0.505	0.693 (0.308-1.559)	0.375
qSOFA	1.647 (1.019-2.662)	0.041	1.934 (1.219-3.071)	0.005
Diabetes	2.303 (0.932-5.688)	0.071	1.902 (0.822-4.398)	0.133
Renal disease	9.394 (1.082-81.587)	0.042	13.333 (1.598-111.232)	0.017
log__10__ (mIBI)	2.771 (1.920-3.999)	<0.001	/	/
PCT/LYM	/	/	1.045 (1.016-1.076)	0.002
AUC	0.893		0.859	
Nagelkerke R²	0.519		0.464	
H-L *P*	0.129		0.939	

Model 1 = age, sex, qSOFA, diabetes, renal insufficiency + log_10_ (mIBI); Model 2 = age, sex, qSOFA, diabetes, renal insufficiency + PCT/LYM. LYM was not included as a covariate because it is a denominator component of both composite indices. mIBI, modified Inflammatory Burden Index (PCT × CRP/LYM); PCT, Procalcitonin; LYM, Lymphocyte Count; qSOFA, Quick Sequential Organ Failure Assessment.

In the septic shock diagnostic model (**Table 4**, panel B, Sepsis Group vs Septic Shock Group, n = 122), log_10_(mIBI) achieved statistical significance as an independent predictor of shock (OR = 1.399, 95% CI: 1.033–1.894, P = 0.030). In the model where PCT/LYM was substituted for mIBI, PCT/LYM showed a borderline association that did not reach conventional statistical significance (OR = 1.004, 95% CI: 1.000–1.008, P = 0.053). The overall discriminative performance of the two models was comparable (Model B1 AUC = 0.721 vs. Model B2 AUC = 0.731). Sensitivity analyses excluding qSOFA yielded a similar comparative pattern (mIBI: OR = 1.422, 95% CI: 1.063–1.901, P = 0.018, AUC = 0.668; PCT/LYM: OR = 1.004, 95% CI: 1.000–1.008, P = 0.048, AUC = 0.678), although absolute AUCs decreased as expected when the clinical severity covariate was removed. Supplementary models additionally adjusting for LYM produced consistent directionality (mIBI: OR = 1.580, P = 0.009; PCT/LYM: OR = 1.005, P = 0.027). The borderline significance of PCT/LYM in the primary model and the modest AUC values likely reflect the limited sample size in this subgroup (n = 122) and the pathophysiological complexity of the sepsis-to-shock transition, and these findings warrant further validation in larger prospective cohorts.

**Table 4:** Multivariate logistic regression analysis for the diagnosis of septic shock (sepsis group vs septic shock group, n = 122).

Variable	Model 1 OR (95% CI)	Model 1 *P*	Model 2 OR (95% CI)	Model 2 *P*
Age	1.021 (0.988-1.054)	0.217	1.020 (0.987-1.054)	0.233
Female (vs Male)	0.565 (0.230-1.391)	0.214	0.597 (0.246-1.449)	0.254
qSOFA	1.942 (1.243-3.034)	0.004	1.963 (1.269-3.036)	0.002
Diabetes	0.786 (0.336-1.839)	0.579	0.758 (0.325-1.767)	0.521
Renal disease	1.260 (0.465-3.419)	0.649	1.421 (0.526-3.837)	0.488
log__10__ (mIBI)	1.399 (1.033-1.894)	0.030	/	/
PCT/LYM	/	/	1.004 (1.000-1.008)	0.053
AUC	0.721		0.731	
Nagelkerke R²	0.220		0.221	
H-L *P*	0.093		0.042	

Model 1, age, sex, qSOFA, diabetes, renal insufficiency + log_10_ (mIBI); Model 2 = age, sex, qSOFA, diabetes, renal insufficiency + PCT/LYM. LYM was not included as a covariate because it is a denominator component of both composite indices. mIBI, modified Inflammatory Burden Index (PCT × CRP/LYM); PCT, Procalcitonin; LYM, Lymphocyte Count; qSOFA, Quick Sequential Organ Failure Assessment.

### 3.4 Analysis of diagnostic efficacy in the sepsis group

Using patients with non-septic infectious inflammation as the control group, the diagnostic performance of the mIBI, conventional biomarkers, and composite indicators for sepsis was evaluated using ROC curve analysis (see **Table 5**; **Figure 2**). Among all evaluated indicators, PCT/LYM demonstrated strong diagnostic performance, with an AUC of 0.851 (95% CI: 0.775-0.928, *p* < 0.001). At the optimal cutoff value of 0.91, the sensitivity and specificity were 80.00% and 81.48%, respectively, with a positive predictive value (PPV) of 78.26% and a negative predictive value (NPV) of 83.02%, indicating a favorable balance between diagnostic accuracy and the risk of missed diagnosis. Bootstrap internal validation with 1,000 resamples confirmed the stability of this cutoff (95% CI: 0.41-2.20), which encompassed the original value. mIBI also exhibited strong diagnostic performance, with an AUC of 0.806 (95% CI: 0.718-0.894, *p* < 0.001).

**Table 5:** Diagnostic performance of biomarkers for sepsis.

Indicator	Optimal cutoff value	AUC	95%CI	*P*	Sensitivity (%)	Specificity (%)	PPV (%)	NPV (%)	Bootstrap cutoff 95% CI
PCT	0.99ng/mL	0.841	0.760~0.921	0.000	80.00	85.19	81.82	83.64	0.539~1.330
CRP	95.63mg/L	0.597	0.481~0.712	0.099	55.56	72.22	62.50	66.10	72.07~279.98
IL-6	281.20pg/mL	0.864	0.792~0.936	0.000	71.11	88.89	84.21	78.69	92.71~533.90
NEU	8.91×10^9^/L	0.603	0.491~0.716	0.077	66.67	59.26	57.69	68.09	5.71~13.56
LYM	0.75×10^9^/L	0.740	0.638~0.840	0.000	77.78	66.67	66.04	75.51	0.580~1.080
mIBI	328.33	0.806	0.718~0.894	0.000	64.44	88.89	82.86	75.00	17.49~495.67
CRP/LYM	126.37	0.671	0.560~0.781	0.004	62.22	74.07	66.67	70.18	70.16~332.37
PCT/LYM	0.91	0.851	0.775~0.928	0.000	80.00	81.48	78.26	83.02	0.409~2.217
NLR	7.50	0.743	0.647~0.840	0.000	91.11	50.00	60.29	87.10	7.52~17.30
qSOFA	≥2	0.540	0.431~0.643	0.464	24.44	81.48	52.38	56.41	/

**Figure 2:**
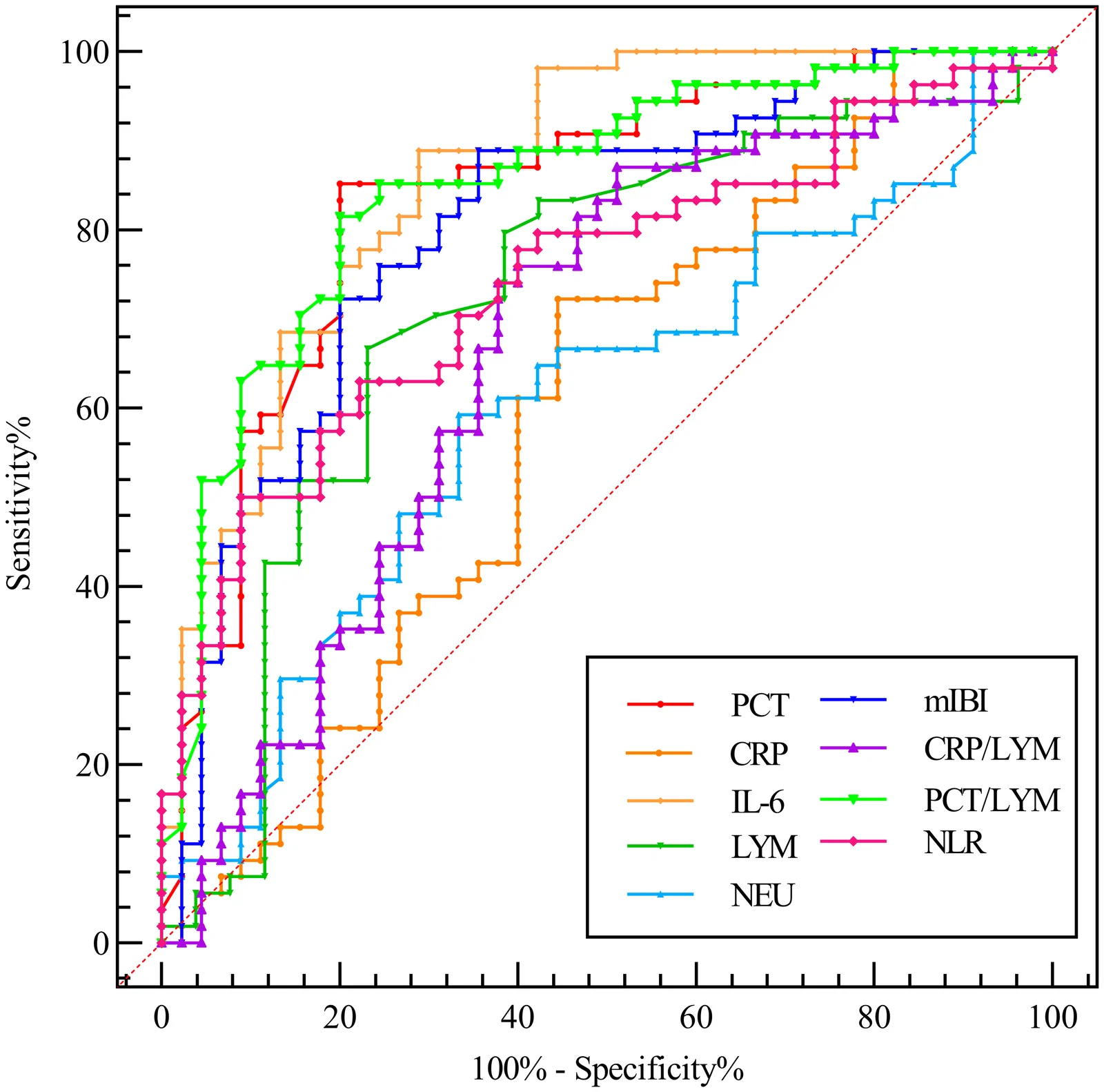
ROC curves for biomarkers in the diagnosis of sepsis.

At a cutoff value of 328.33, the specificity and sensitivity were 88.89% and 64.44%, respectively, yielding a PPV of 82.86% and an NPV of 75.00%, indicating a particular advantage in minimizing false-positive results and suitability for clinical contexts requiring high specificity. Bootstrap validation confirmed the stability of the mIBI cutoff (95% CI: 17.65-495.67). Among conventional biomarkers, IL-6 demonstrated the highest diagnostic performance (AUC = 0.864, 95% CI: 0.792-0.936, *p* < 0.001), with sensitivity and specificity of 71.11% and 88.89%, respectively, and the highest PPV among all indicators (84.21%).

In comparison, PCT demonstrated an AUC of 0.841 (95% CI: 0.760-0.921, *p* < 0.001) with a PPV of 81.82% and an NPV of 83.64%, whereas CRP demonstrated a markedly lower AUC of 0.597 (95% CI: 0.481-0.712, *P =* 0.099), indicating limited diagnostic utility. Among composite indicators, NLR exhibited the highest sensitivity (91.11%) but relatively low specificity (50.00%), resulting in a PPV of only 60.29% despite the highest NPV (87.10%). CRP/LYM demonstrated moderate diagnostic performance, with an AUC of 0.671 (95% CI: 0.560-0.781, *P =* 0.004), a PPV of 66.67%, and an NPV of 70.18%. Bootstrap internal validation confirmed the stability of AUC values for all indicators (**Table 5**). ROC curve analysis (**Figure 2**) indicated that the curves for PCT/LYM, mIBI, and IL-6 were closest to the upper left corner, reflecting favorable diagnostic efficacy.

DeLong’s test for paired correlated ROC areas (**Table 6**, Panel A) further revealed that mIBI was significantly superior to CRP (ΔAUC = 0.209, Z = 4.825, *P* < 0.001), NEU (ΔAUC = 0.202, Z = 3.136, *P* = 0.002), CRP/LYM (ΔAUC = 0.135, Z = 3.741, *P* < 0.001), and PCT/LYM (ΔAUC = −0.045, Z = −2.055, *P* = 0.040) in distinguishing sepsis patients from controls. No statistically significant differences were observed between mIBI and PCT (*P* = 0.186), IL-6 (*P* = 0.306), LYM (*P* = 0.158), or NLR (*P* = 0.140). Notably, although PCT/LYM showed a nominally higher AUC than mIBI (0.851 vs. 0.806), the difference did not reach the Bonferroni-corrected significance threshold (adjusted α = 0.05/8 = 0.00625; the eight pre-specified comparisons per panel were mIBI vs. PCT, CRP, IL-6, LYM, NEU, CRP/LYM, PCT/LYM, and NLR; no all-pairs comparisons among all nine biomarkers were performed), indicating comparable discriminative ability between the two. The direct DeLong comparison between PCT/LYM and PCT was not statistically significant (AUC 0.851 vs. 0.841, ΔAUC = 0.010, Z = −0.762, P = 0.446). In the present cohort, qSOFA yielded an AUC of 0.540 (95% CI: 0.431–0.643, P = 0.464) for sepsis diagnosis; at the prespecified cutoff of ≥2, sensitivity was 24.44%, specificity 81.48%, PPV 52.38%, and NPV 56.41%, indicating limited discriminative ability. This AUC was significantly lower than both mIBI (DeLong Z = −4.013, P < 0.001) and PCT/LYM (Z = −4.706, P < 0.001).

**Table 6:** DeLong test for pairwise comparison of AUCs.

A. Sepsis vs control (n=45 vs n=54)						
Comparison	AUC (primary)		AUC (comparator)	ΔAUC	*Z*	*P*
mIBI vs PCT	0.806		0.841	-0.035	-1.323	0.186
mIBI vs CRP	0.806		0.597	0.209	4.825	<0.001 ***
mIBI vs IL-6	0.806		0.864	-0.058	-1.024	0.306
mIBI vs NEU	0.806		0.603	0.202	3.136	0.002 **
mIBI vs LYM	0.806		0.739	0.067	1.412	0.158
mIBI vs CRP/LYM	0.806		0.671	0.135	3.741	<0.001 ***
mIBI vs PCT/LYM	0.806		0.851	-0.045	-2.055	0.040 *
mIBI vs NLR	0.806		0.743	0.063	1.477	0.140
PCT/LYM vs PCT	0.851		0.841	0.010	-0.762	0.446
qSOFA vs mIBI	0.540		0.806	-0.266	-4.013	<0.001 ***
qSOFA vs PCT/LYM	0.540		0.851	-0.311	-4.706	<0.001 ***
B. Septic shock vs control (n=77 vs n=54)						
mIBI vs PCT	0.877	0.879		-0.002	-0.110	0.913
mIBI vs CRP	0.877	0.777		0.100	3.574	<0.001 ***
mIBI vs IL-6	0.877	0.879		-0.002	-0.073	0.942
mIBI vs NEU	0.877	0.568		0.309	5.821	<0.001 ***
mIBI vs LYM	0.877	0.674		0.203	4.690	<0.001 ***
mIBI vs CRP/LYM	0.877	0.768		0.109	3.819	<0.001 ***
mIBI vs PCT/LYM	0.877	0.880		-0.003	-0.231	0.818
mIBI vs NLR	0.877	0.682		0.195	4.819	<0.001 ***
PCT/LYM vs PCT	0.880	0.879		0.001	-0.124	0.902
qSOFA vs mIBI	0.719	0.877		-0.158	-2.951	0.003 **
qSOFA vs PCT/LYM	0.719	0.880		-0.161	-3.020	0.003 **

DeLong test for paired comparison of correlated ROC areas from the samesamples. ΔAUC, AUC(primary indicator) − AUC(comparator). For the primary 8comparisons per panel, the primary indicator is mIBI, and the Bonferroni-correctedthreshold was α = 0.05/8 = 0.00625. *Post hoc* comparisons (PCT/LYM vs. PCT andqSOFA comparisons) were considered exploratory and were not included in theBonferroni-corrected family. *P < 0.05, **P < 0.01, ***P < 0.001.

### 3.5 Analysis of diagnostic efficacy in the septic shock group

Using patients with non-septic infectious inflammation as the control group, the diagnostic performance of various indicators for septic shock was evaluated (see **Table 7**; **Figure 3**). The results indicated that PCT/LYM achieved the numerically highest AUC of 0.880 (95% CI: 0.823-0.936, *p* < 0.001).

**Table 7:** Diagnostic performance of biomarkers for septic shock.

Indicator	Optimal cutoff value	AUC	95% CI	*P*	Sensitivity (%)	Specificity (%)	PPV (%)	NPV (%)	Bootstrap cutoff 95% CI
PCT	0.95ng/mL	0.879	0.821~0.936	0.000	84.42	83.33	87.84	78.95	0.973~8.990
CRP	100.49mg/L	0.777	0.698~0.855	0.000	75.32	72.22	79.45	67.24	62.50~258.06
IL-6	105.65pg/mL	0.879	0.821~0.937	0.000	88.31	72.22	81.93	81.25	76.79~598.60
NEU	11.17×10^9^/L	0.568	0.470~0.666	0.184	53.25	68.52	70.69	50.68	7.66~22.92
LYM	0.71×10^9^/L	0.674	0.581~0.767	0.001	64.94	72.22	76.92	59.09	0.530~0.840
mIBI	99.78	0.877	0.819~0.935	0.000	80.52	81.48	86.11	74.58	30.22~702.31
CRP/LYM	159.83	0.768	0.686~0.849	0.000	67.53	81.48	83.87	63.77	56.93~337.24
PCT/LYM	1.61	0.880	0.823~0.936	0.000	76.62	85.19	88.06	71.88	0.656~8.368
NLR	15.44	0.681	0.589~0.774	0.000	62.34	77.78	80.00	59.15	9.36~19.39
qSOFA	≥2	0.719	0.632~0.802	<0.001	49.35	81.48	79.17	53.01	/

**Figure 3:**
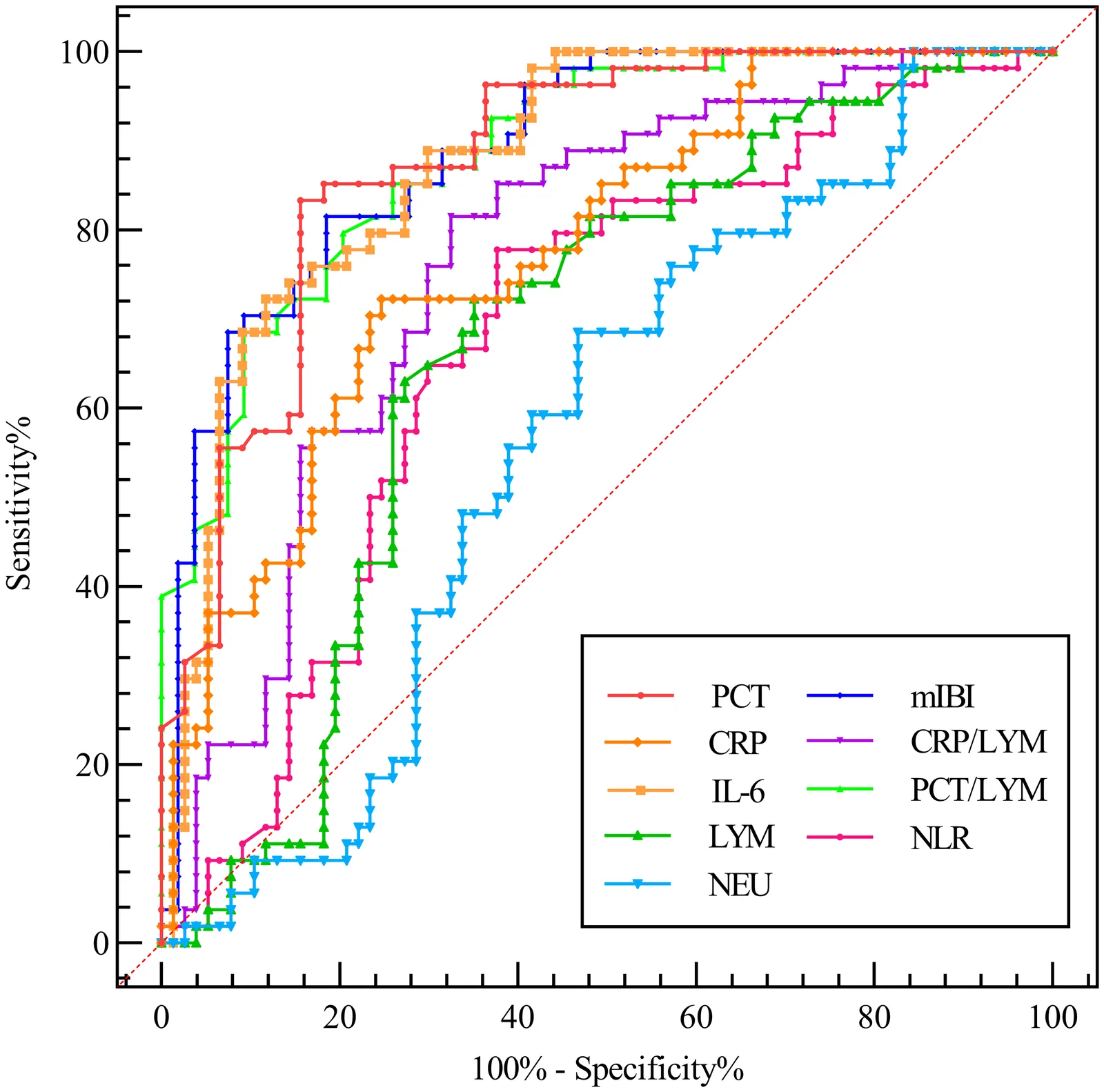
ROC curves for biomarkers in the diagnosis of septic shock.

At the optimal cutoff value of 1.61, the sensitivity and specificity were 76.62% and 85.19%, respectively, with a PPV of 88.06% and an NPV of 71.88%, indicating its effectiveness in identifying patients progressing to septic shock. Bootstrap internal validation with 1,000 resamples confirmed the stability of this cutoff (95% CI: 0.66-8.37). The diagnostic performance of mIBI was further enhanced, with an AUC of 0.877 (95% CI: 0.819-0.935, *p* < 0.001). At a cutoff value of 99.78, the sensitivity and specificity were 80.52% and 81.48%, respectively, yielding a PPV of 86.11% and an NPV of 74.58%, indicating improved sensitivity while maintaining high specificity, thereby supporting its utility for early prediction of septic shock. The Bootstrap 95% CI for the mIBI cutoff was 30.22-702.31. Among conventional biomarkers, both PCT and IL-6 demonstrated comparable diagnostic performance, each with an AUC of 0.879 (95% CI: 0.821-0.936/0.937, *p* < 0.001). PCT/LYM exhibited the highest PPV (88.06%) among all indicators, while PCT showed a PPV of 87.84% and an NPV of 78.95%. IL-6 demonstrated a PPV of 81.93% and an NPV of 81.25%.

Compared with findings in the sepsis group, CRP demonstrated a notable improvement in diagnostic performance in the septic shock group, with an AUC of 0.777 (95% CI: 0.698-0.855, *p* < 0.001), a PPV of 79.45%, and an NPV of 67.24%. Among composite indicators, CRP/LYM yielded an AUC of 0.768 (95% CI: 0.686-0.849, *p* < 0.001) with a PPV of 83.87% and an NPV of 63.77%, while NLR demonstrated an AUC of 0.681 (95% CI: 0.589-0.774, *p* < 0.001) with a PPV of 80.00% and an NPV of 59.15%. Bootstrap internal validation confirmed the stability of AUC values for all indicators (**Table 7**). ROC curve analysis (**Figure 3**) demonstrated that the curves for mIBI, PCT/LYM, PCT, and IL-6 were closely aligned and demonstrated superior performance compared with those of CRP, LYM, and NEU.

DeLong’s test (**Table 6**, Panel B) demonstrated that mIBI was significantly superior to CRP (ΔAUC = 0.100, Z = 3.574, *P* < 0.001), NEU (ΔAUC = 0.309, Z = 5.821, *P* < 0.001), LYM (ΔAUC = 0.203, Z = 4.690, *P* < 0.001), CRP/LYM (ΔAUC = 0.109, Z = 3.819, *P* < 0.001), and NLR (ΔAUC = 0.195, Z = 4.819, *P* < 0.001) for predicting septic shock. No significant differences were found between mIBI and PCT (*P* = 0.913), IL-6 (*P* = 0.942), or PCT/LYM (*P* = 0.818), confirming that mIBI performed comparably to PCT and PCT/LYM, two of the strongest individual predictors for shock risk stratification. The direct DeLong comparison between PCT/LYM and PCT was likewise not statistically significant (AUC 0.880 vs. 0.879, ΔAUC = 0.001, Z = -0.124, P = 0.902). For septic shock prediction, qSOFA yielded an AUC of 0.719 (95% CI: 0.632–0.802, P < 0.001); at the prespecified cutoff of ≥2, sensitivity was 49.35%, specificity 81.48%, PPV 79.17%, and NPV 53.01%. This AUC was significantly lower than both mIBI (DeLong Z = 2.951, P = 0.003) and PCT/LYM (Z = 3.020, P = 0.003).

## 4 Discussion

Sepsis and septic shock, as progressive stages of the same pathological process, remain major challenges in global emergency and critical care medicine, with high morbidity and mortality rates largely attributable to delays in early identification and imprecise disease stratification (Tidswell et al., 2021). Although diagnostic criteria have been established in the Sepsis-3 international consensus, reliance on single biomarkers (e.g., PCT, CRP) or conventional clinical scoring systems (e.g., qSOFA) remains insufficient to comprehensively capture the complex pathophysiological continuum of sepsis, characterized by infection activation, inflammatory response amplification, and immune suppression (Seymour et al., 2016; Shappell et al., 2023).

Biomarkers therefore play a critical role in improving diagnostic accuracy. Traditional biomarkers, including PCT, CRP, and IL-6, are widely used in the evaluation of bacterial infections; however, their clinical utility is limited in distinguishing sepsis from other inflammatory conditions and in predicting disease progression (Chen et al., 2025). The inflammatory response induced by tissue injury closely resembles that observed in sepsis, and similar alterations in these biomarkers may also occur in SIRS (Schlattmann and Brunkhorst, 2014; Chen et al., 2025). CRP and PCT are acute-phase proteins, with CRP production stimulated by IL-6 in the liver in response to tissue injury, inflammation, or infection. CRP levels typically increase within 4–6 hours following stimulation, double within approximately 8 hours, and peak between 36 and 50 hours, supporting its role as an indicator of disease severity (Katsaros et al., 2022; Juneja et al., 2023; Rizo-Téllez et al., 2023). Peripheral lymphocytopenia is a hallmark of immunosuppression in sepsis, and patients with persistent lymphocyte counts < 0.76 × 10^9^/L have a 28-day mortality rate of 39.66%, significantly higher than that of patients without persistent lymphocytopenia (Tocu et al., 2023).

A recent study reported that lymphocyte count demonstrated a diagnostic sensitivity of 85.4% for sepsis and 90.5% for pulmonary sepsis; however, its specificity was limited (61.4%) in the diagnosis of pulmonary sepsis (Liu et al., 2022), indicating that it should be combined with other biomarkers to improve diagnostic specificity. Multiple biomarkers are therefore used in clinical practice to assess immune status in patients with sepsis. The NLR, derived from routine blood testing, serves as both an inflammatory marker and an independent risk factor, as well as an important predictor of prognosis (Zheng et al., 2024; Chen et al., 2025). Key pathophysiological features of sepsis include lymphocyte apoptosis and dysfunction leading to immunosuppression, impaired secretion of pro-inflammatory cytokines, and expansion of regulatory immune cells accompanied by increased release of anti-inflammatory cytokines (Li et al., 2025).

Although previous studies have evaluated the diagnostic value of individual biomarkers for sepsis, limited evidence is available regarding the utility of the PCT/LYM and the modified IBI (mIBI = PCT × CRP/LYM) for early identification of sepsis and prediction of septic shock. To the best of our knowledge, extensive data search reveals no prior systematic evaluation of these specific composite indices for sepsis stratification. The present study provides a systematic evaluation of these two indicators. The findings demonstrated that, in the diagnostic analysis of sepsis, PCT/LYM exhibited strong diagnostic performance, with an AUC of 0.851 (95% CI: 0.775-0.928). At an optimal cutoff value of 0.91, the sensitivity and specificity were 80.00% and 81.48%, respectively, outperforming traditional biomarkers such as CRP (AUC = 0.597) and NLR (AUC = 0.743), and demonstrating comparable performance to IL-6 (AUC = 0.864). This advantage may be attributed to the integrated assessment of infection burden and immune status. PCT serves as a relatively specific biomarker for bacterial infection, with levels reflecting infection severity and minimal interference from non-infectious factors such as viral infections and autoimmune conditions.

Previous studies have reported that the specificity of PCT for diagnosing bacterial infection can reach 92.3%, which is higher than that of CRP (78.5%) (Leung et al., 2024). LYM, as a key indicator of immune function, is inversely associated with the degree of immunosuppression in sepsis and has demonstrated value in predicting disease progression (B and Yang, 2026). In contrast, although mIBI exhibited high specificity (88.89%), its lower sensitivity (64.44%) indicates greater applicability in clinical scenarios requiring high specificity for exclusion (e.g., reducing overtreatment in suspected cases). PCT/LYM, which provides a more balanced sensitivity and specificity profile, may therefore be a useful candidate for early sepsis screening as part of a composite approach. These findings are consistent with those of a previous small-sample study (AUC = 0.859), further supporting the stability and reliability of this indicator (Gao et al., 2025).

Regarding the early predictive performance for septic shock, the present study demonstrated that PCT/LYM (AUC = 0.880) and mIBI (AUC = 0.877) exhibited strong diagnostic accuracy, comparable to that of PCT and IL-6 (all AUC = 0.879), and superior to CRP (AUC = 0.777). These findings support the conclusion that composite indicators can achieve diagnostic performance comparable to established biomarkers for early prediction of septic shock while offering enhanced clinical applicability. Notably, the optimal cutoff value of PCT/LYM for shock diagnosis (1.61) was markedly higher than that for sepsis diagnosis, reflecting the progressive increase in infection burden and the exacerbation of immune suppression during the transition from sepsis to septic shock. The dynamic changes in this ratio provide a quantitative indicator of disease progression, indicating that serial monitoring of PCT/LYM may facilitate early identification of patients at risk of developing septic shock. This observation is consistent with previous findings reported by Tian et al. (2021).

The early predictive value of mIBI may be attributed to its ability to capture the pathophysiological cascade involving cytokine storm, lymphocyte apoptosis, and subsequent immune dysfunction, with a sensitivity of 80.52% and specificity of 81.48% at a cutoff value of 99.78. This enables earlier identification of high-risk patients prior to the onset of overt clinical manifestations of shock, thereby providing a critical window for timely interventions, such as fluid resuscitation and administration of vasoactive agents.

In a *post hoc* ROC analysis within the present cohort, qSOFA at a cutoff of ≥2 yielded limited discriminative ability for sepsis (AUC = 0.540, 95% CI 0.431–0.643; sensitivity 24.44%, specificity 81.48%) and only moderate discrimination for septic shock (AUC = 0.719, 95% CI 0.632–0.802; sensitivity 49.35%, specificity 81.48%). Both composite indices, PCT/LYM and mIBI, significantly outperformed qSOFA in head-to-head DeLong comparisons (P < 0.001 for Panel A and P = 0.003 for Panel B), highlighting the additional discriminative value of laboratory-based composites over the bedside score (Monaco et al., 2020). Furthermore, the present study demonstrated that the diagnostic performance of CRP improved in the septic shock group compared with the sepsis group (AUC increased from 0.597 to 0.777), indicating that its value in reflecting inflammatory progression becomes more pronounced as disease severity increases. However, CRP should be used in combination with composite indicators such as mIBI or PCT/LYM to enhance diagnostic accuracy and reduce false-positive results associated with non-infectious inflammatory conditions (Liu et al., 2024).

In the present study, based on routine hematological parameters combined with CRP and PCT, mIBI demonstrated a higher AUC for predicting septic shock (AUC = 0.877) compared with the qSOFA score in the present cohort (AUC = 0.719 for septic shock; P = 0.003 by DeLong test), while its AUC for sepsis was 0.806 versus 0.540 for qSOFA (P < 0.001) (Raith et al., 2017). The mIBI was originally proposed by Xie et al. (2022) as CRP × NEU/LYM in a multicenter cancer cohort, aiming to quantify the systemic inflammatory burden. In the present study, the formula was adapted to PCT × CRP/LYM, substituting NEU with PCT-a biomarker with demonstrated specificity for bacterial infection (Ito et al., 2022)-thereby redirecting the index from a general inflammatory marker to one that integrates infection burden, acute-phase response, and immune status. Multivariable logistic regression confirmed that both mIBI and PCT/LYM were independent predictors of sepsis after adjustment for age, sex, qSOFA, diabetes, and renal insufficiency. Supplementary models additionally adjusting for LYM produced consistent findings, with LYM itself non-significant in both models. This indicates that although LYM constitutes a shared component of both formulas, it does not act as a confounder in the primary models; rather, mIBI captures multidimensional pathophysiological information-encompassing the neutrophil-to-lymphocyte ratio, platelet-to-lymphocyte ratio, and systemic immune-inflammation index (SII)-that reflects the simultaneous dysregulation of infection, inflammation, coagulation, and immunity characteristic of sepsis (Gao et al., 2025). This interpretation is supported by a recent meta-analysis of 25,626 sepsis patients (Liang and Su, 2025), which demonstrated that SII, a related composite index sharing the NEU/LYM and platelet/lymphocyte dimensions with mIBI, was independently associated with short-term mortality (pooled RR = 1.51, 95% CI: 1.31-1.67). Notably, the model incorporating log__10__(mIBI) yielded a higher AUC (0.893) than that incorporating PCT/LYM (0.859), suggesting that the additional inflammatory and coagulation dimensions captured by mIBI may provide incremental diagnostic value. Importantly, mIBI requires no additional testing beyond routine hematological parameters and PCT, making it particularly suitable for resource-limited settings.

In the septic shock analysis, mIBI retained independent predictive capacity (OR = 1.399, P = 0.030), whereas PCT/LYM showed only a borderline association (P = 0.053). This likely reflects the shift in dominant pathophysiology at the shock stage and the limited sample size of this subgroup (n = 122). Septic shock involves a biphasic immune response—an initial hyperinflammatory phase followed by a compensatory immunosuppressive phase characterized by profound lymphopenia driven by lymphocyte apoptosis via both the Fas/FasL extrinsic and mitochondrial intrinsic pathways (Gao et al., 2025), and upregulation of immune checkpoint molecules including PD-1/PD-L1 (Monaco et al., 2020). Sensitivity analyses without qSOFA confirmed that the comparative pattern between mIBI and PCT/LYM was stable, although absolute discrimination decreased as expected. The borderline significance of PCT/LYM and the relatively wide confidence intervals in this subgroup suggest that larger cohorts are needed to precisely estimate the independent contributions of these composite indices in septic shock. Taken together, these results support mIBI and PCT/LYM as complementary biomarkers: mIBI excels in sepsis diagnosis by integrating multidimensional inflammatory information with stronger independent effect sizes, whereas PCT/LYM demonstrates comparable univariate discrimination and may offer practical advantages due to its simpler formula.

Several limitations of the present study warrant acknowledgment. First, this was a single-center retrospective study with a relatively small sample size, which may limit the generalizability of the findings and the statistical power of subgroup analyses, particularly for the septic shock subgroup in multivariable modeling. Second, although bootstrap resampling (1000 iterations) was employed for internal validation, the relatively wide confidence intervals of some AUC estimates suggest potential overfitting, and external validation in independent cohorts is needed. Third, the exact timing of blood sample collection relative to admission was not uniformly documented, although all samples were obtained within 24 hours of admission and prior to antibiotic administration in most cases. Fourth, qSOFA was retained as a clinical severity covariate because it is assessed at presentation before diagnosis; sensitivity analyses excluding qSOFA yielded the same comparative pattern between mIBI and PCT/LYM, although absolute AUCs were lower, indicating that the biomarker effects were not artifacts of over-adjustment. Fifth, the lack of statistically significant difference between PCT/LYM and PCT in DeLong testing indicates that the nominally higher AUC of PCT/LYM should be interpreted as a numerical improvement rather than a demonstrated superiority. Sixth, severity scoring systems including SOFA, APACHE II, and NEWS2 were not systematically recorded, precluding direct comparison with these established prognostic tools; future studies should incorporate such scores to enable comprehensive benchmarking. Seventh, the pathogen spectrum was not stratified in the subgroup analyses, and the potential influence of specific pathogens on biomarker levels remains to be elucidated. The predominance of Gram-negative bacteria in our cohort (≈78%) limits generalizability to Gram-positive or fungal sepsis, where biomarker kinetics may differ. Finally, although rigorous retrospective verification was performed for the control group-including confirmation of negative blood cultures, PCT below the exclusion threshold, and review of complete hospitalization trajectories by attending physicians-the possibility of misclassification of patients in early, undocumented sepsis cannot be entirely excluded.

PCT/LYM may serve as a primary indicator for early screening of sepsis due to its balanced sensitivity and specificity, whereas mIBI may be preferentially applied as an early warning indicator for septic shock. In addition, CRP/LYM may function as a cost-effective tool for excluding septic shock in primary care settings (specificity: 81.48%). The combined application of these indicators enables comprehensive coverage across the clinical continuum, from early identification to risk stratification, thereby addressing challenges related to delayed recognition and inadequate disease stratification. Furthermore, the distinct cutoff values of PCT/LYM for sepsis and septic shock (0.91 vs. 1.61) provide a quantitative representation of the dynamic progression of infection burden and immune suppression, supporting the use of serial monitoring of this single indicator to assess the risk of disease progression.

## 5 Conclusion

The composite indicator framework integrating PCT/LYM and mIBI addresses the limitations associated with single biomarkers and enables a comprehensive approach to sepsis management. PCT/LYM demonstrated high univariate diagnostic accuracy and independent predictive value for sepsis, with numerically higher AUC than PCT alone for both sepsis and septic shock, although the direct DeLong comparison did not reach statistical significance. mIBI exhibited strong independent diagnostic value for both sepsis (OR = 2.771, P < 0.001) and septic shock (OR = 1.399, P = 0.030) after adjustment for clinical confounders. Sensitivity analyses excluding qSOFA confirmed the robustness of these findings. The combined application of these indicators, based on routinely available laboratory parameters, offers advantages in timeliness and cost-effectiveness, particularly in primary healthcare settings, and may contribute to improved early identification and risk stratification of sepsis.

## Data Availability

The original contributions presented in the study are included in the article/supplementary material. Further inquiries can be directed to the corresponding author.
